# Pulmonary toxicity of craniospinal irradiation using helical tomotherapy

**DOI:** 10.1038/s41598-022-07224-1

**Published:** 2022-02-25

**Authors:** Joongyo Lee, Euidam Kim, Nalee Kim, Chang-Ok Suh, Yoonsun Chung, Hong In Yoon

**Affiliations:** 1 Department of Radiation Oncology, Yonsei Cancer Center, Yonsei University Health System, Yonsei University College of Medicine, 50-1 Yonsei-ro, Seodaemun-gu, Seoul, 03722 Republic of Korea; 2 Department of Nuclear Engineering, Hanyang University, 222 Wangsimni-ro, Seongdong-gu, Seoul, 04763 Republic of Korea; 3 Department of Radiation Oncology, Samsung Medical Center, Sungkyunkwan University School of Medicine, Seoul, Republic of Korea; 4 Department of Radiation Oncology, Bundang CHA Medical Center, CHA University, Gyeonggi-do, Republic of Korea

**Keywords:** Radiotherapy, CNS cancer

## Abstract

Craniospinal irradiation using helical tomotherapy (HT-CSI) has advantages in aspects of homogeneous dose distribution. Physicians, however, still have concerns of pulmonary toxicity due to HT-CSI’s relatively large, low-dose irradiated volume from continuous and 360° rotation delivery. In this study, we investigated the pulmonary toxicity of HT-CSI. We retrospectively reviewed 105 patients who received HT-CSI between January 2014 and December 2019. Grade 2 + pulmonary toxicities were evaluated. Intensive systemic treatment was defined as systemic treatment administration before, during, and after HT-CSI. V_X Gy_ was defined as % volume receiving ≥ X Gy. Thirteen patients (12.4%) presented with grade 2 + pulmonary toxicities after HT-CSI. Of these patients, only one experienced grade 2 radiation pneumonitis combined with pembrolizumab-induced pneumonitis. Conversely, pneumonia was observed in 12 patients. Intensive systemic treatment (*p* = 0.004), immunosuppressive drugs (*p* = 0.031), and bilateral lung V_5 Gy_ ≥ 65% (*p* = 0.031) were identified as independent risk factors for pneumonia. The risk factor for pneumonia in pediatric patients were immunosuppressive drugs (*p* = 0.035) and bilateral lung V_5 Gy_ ≥ 65% (*p* = 0.047). HT-CSI can be a safe treatment modality with tolerable pulmonary toxicities. Intensive systemic treatment, immunosuppressive drugs, and bilateral lung V_5 Gy_ ≥ 65% were significantly associated with pneumonia. In these patients, close follow-up should be considered for proper management of pneumonia.

## Introduction

Craniospinal irradiation (CSI) is necessary for preventing the spread of central nervous system tumors via cerebrospinal fluid, or for palliative purposes when leptomeningeal seeding of solid cancers causes neurologic symptoms^1–3^.

Yonsei Cancer Center has conducted CSI using two-dimensional radiotherapy (RT) from 1992 and three-dimensional conformal RT (3D-CRT) from 2000. However, due to the low conformity of the 3D-CRT itself, it has the disadvantage of releasing unnecessary doses to the normal tissue and organs around the target^4,5^. This is especially highlighted in children. The main patients receiving CSI are children, and they are known to have more serious side effects: endocrine system disorders, growth disorders, and secondary malignancy after RT^6,7^.

Since 2012, our institution has compensated for this disadvantage of 3D-CRT by setting up CSI using helical tomotherapy (HT-CSI), and helical tomotherapy (HT) is still used as the main modality of CSI. HT-CSI generally gives a more conformal dose distribution than 3D-CRT. Thus, unnecessary doses irradiated to normal tissues or organs near the target can be reduced, thereby reducing side effects^8,9^. However, HT-CSI has potential limits. It is characterized by a full 360-degree delivery, which is continuously irradiated in all directions. It has a wider low dose distribution than that in 3D-CRT for CSI (see Supplementary Fig. S1 online). For this reason, there is a concern about pulmonary toxicity caused by HT-CSI^10,11^.

In this study, we analyzed the prevalence and risk factors for pulmonary toxicity in patients receiving HT-CSI and ultimately investigated the safety and feasibility of HT-CSI. In addition, we tried to determine whether a difference exists between pediatric and adult patients with pulmonary toxicity due to HT-CSI.

## Results

### Patient, treatment, and dosimetric characteristics

The baseline characteristics of patients are summarized in Table 1. There were 51 pediatric (48.6%) and 54 adult patients (51.4%). The median age at CSI was 21 years (range 2–74). The most common disease was germ cell tumor in pediatric (22/51, 43.1%) and glioblastoma in adult (14/54, 25.9%) patients, respectively.

**Table 1:** Patient characteristics of total patients and patients categorized by age of 20.

Characteristic	Total (N = 105)		Pediatric (N = 51)		Adult (N = 54)	
	N	%	N	%	N	%
**Age (years)**						
Median (range)	21 (2–74)		11 (2–19)		46 (20–74)	
**Sex**						
Male	62	59.0	32	62.7	30	55.6
Female	43	41.0	19	37.3	24	44.4
**Histology**						
Germ cell tumor	27	25.7	22	43.1	5	9.3
Medulloblastoma	18	17.2	15	29.4	3	5.5
Glioblastoma	14	13.3	0	0.0	14	25.9
Solid tumor—Leptomeningeal carcinomatosis	10	9.5	1	2.0	9	16.7
Leukemia—Central nervous system relapse	7	6.7	5	9.8	2	3.7
Diffuse midline glioma	6	5.7	1	2.0	5	9.3
Miscellaneous	23	21.9	7	13.7	16	29.6

Treatment details are listed in Table 2. Total CSI dose for all patients was median 36.0 Gy (range 12.0–45.0) with a fractional dose of median 1.5 Gy (range 1.2–3.0); 23 patients (21.9%) with < 20 Gy, 27 (25.7%) with 20–36 Gy, and 55 (52.4%) with ≥ 36 Gy, respectively. The total CSI dose for 51 pediatric patients was median 23.4 Gy; 20 (39.2%) with < 20 Gy, 17 (33.3%) with 20–36 Gy, and 14 (27.5%) with ≥ 36 Gy, respectively. The total CSI dose for 54 adult patients was median 36.0 Gy; 3 (5.6%) with < 20 Gy, 10 (18.5%) with 20–36 Gy, and 41 (75.9%) with ≥ 36 Gy, respectively. Boost RT was administered to 85 patients (81.0%), 13 (12.4%) of them on T spine level. Systemic treatment was given to 70 patients (66.7%). Forty-six patients (43.8%) received systemic treatment before HT-CSI, 43 (41.0%) after HT-CSI, and 36 (34.3%) during HT-CSI. Intensive systemic treatment, which involved systemic treatment before, during, and after HT-CSI, was given to 15 patients (14.3%); 14 pediatric (27.5%), and one adult patient (1.9%). Sixty-two patients (59.0%) were hospitalized during HT-CSI for median 38 days. During HT-CSI, 29 patients (27.6%) received immunosuppressive drugs; 18 of whom received dexamethasone and 11, prednisolone. Nine pediatric (17.6%) and 20 adult patients (37.0%) were administered immunosuppressive drugs.

**Table 2:** Treatment characteristics of total patients and patients categorized by age of 20.

Characteristic	Total (N = 105)		Pediatric (N = 51)		Adult (N = 54)	
	N	%	N	%	N	%
**Total CSI dose (Gy)**						
Median (range)	36.0 (12.0–45.0)		23.4 (12.0–45.0)		36.0 (12.0–45.0)	
< 20 Gy	23	21.9	20	39.2	3	5.6
20–36 Gy	27	25.7	17	33.3	10	18.5
≥ 36 Gy	55	52.4	14	27.5	41	75.9
**Total CSI fraction number (fractions)**						
Median (range)	20 (8–30)		13 (8–27)		24 (10–30)	
**Dose per fraction for CSI (Gy)**						
Median (range)	1.5 (1.2–3.0)		1.8 (1.5–2.0)		1.5 (1.2–3.0)	
**Boost RT**						
No boost	20	19.0	6	11.8	14	25.9
T-spine level	13	12.4	3	5.9	10	18.5
Other than T-spine	72	68.6	42	82.4	30	55.6
**Systemic treatment**						
No systemic treatment	35	33.3	10	19.6	25	46.3
Pre-RT systemic treatment	46	43.8	34	66.7	12	22.2
Post-RT systemic treatment	43	41.0	24	47.1	19	35.2
Concurrent systemic therapy with CSI	36	34.3	23	45.1	13	24.1
Intensive systemic treatment	15	14.3	14	27.5	1	1.9
Admission during CSI	62	59.0	29	56.9	33	61.1
Period (days, median [range])	38 (3–70)		36 (3–51)		40 (12–70)	
Immunosuppressive drug during CSI	29	27.6	9	17.6	20	37.0

CSI, craniospinal irradiation; Gy, gray; RT, radiotherapy.

### Pulmonary toxicity

Over a median follow-up duration of 10.5 months (range 0.7–62.1 months), there were 16 patients (15.2%) who presented with pulmonary toxicities after HT-CSI; grade 1 in 3, grade 2 in 11, and grade 3 in 2 patients. None of them experienced grade 4 or 5 toxicity. Of the 13 grade 2 + pulmonary toxicities, one was diagnosed with radiation pneumonitis and 12 with pneumonia. Dosimetric characteristics of patients according to the presence of grade 2 + pulmonary toxicities are shown in Supplementary Table S1 online. Each dosimetric parameter showed no statistical difference depending on whether the patient had grade 2 + pulmonary toxicities, and the bilateral lung V_5 Gy_ showed a high tendency in the group of patients with grade 2 + pulmonary toxicities (not statistically significant). V_X Gy_ was defined as the volume of organs at risk of receiving at least X Gy.

The characteristics of the patients who had grade 2 + pulmonary toxicities are summarized in Table 3. Radiation pneumonitis was diagnosed in a total of 4 patients, 3 had grade 1 and 1 patient, grade 2. Of the 12 patients diagnosed with pneumonia, seven were diagnosed with pneumocystis pneumonia and 5 with bacterial pneumonia.

**Table 3:** Characteristics of patients with grade 2 + pulmonary toxicities.

No	Age	Sex	Tumor histology	Timing of systemic treatment (Based on CSI)	Pulmonary toxicity grade	Classification of pulmonary toxicity	Lung V_5 Gy_ (%)
1	2	M	Atypical teratoid/rhabdoid tumor	Neoadjuvant + Concurrent	2	Bacterial pneumonia	31.0
2	4	F	Medulloblastoma	Adjuvant	2	Bacterial pneumonia	70.2
3	12	M	Non-germinoma germ cell tumor	Neoadjuvant	2	Bacterial pneumonia	91.1
4	49	M	Diffuse midline glioma, H3 K27M-mutant	Concurrent	2	Bacterial pneumonia	86.5
5	4	M	Acute lymphocytic leukemia	Neoadjuvant + Concurrent + Adjuvant	2	Pneumocystis pneumonia	26.2
6	9	F	Medulloblastoma	Neoadjuvant + Concurrent + Adjuvant	2	Pneumocystis pneumonia	71.9
7	9	M	Burkitt lymphoma	Neoadjuvant + Concurrent + Adjuvant	2	Pneumocystis pneumonia	27.3
8	19	M	Anaplastic ependymoma	Neoadjuvant + Concurrent + Adjuvant	2	Pneumocystis pneumonia	99.8
9	48	M	Glioblastoma, IDH-wildtype	None	2	Pneumocystis pneumonia	44.2
10	67	M	Small cell lung cancer	Neoadjuvant + Concurrent + Adjuvant	2	Pneumocystis pneumonia	64.7
11	52	M	Malignant melanoma	Concurrent + Adjuvant	2	Radiation pneumonitis	61.5
12	55	M	Glioblastoma	Adjuvant	3	Bacterial pneumonia	65.6
13	35	F	Mixed oligoastrocytoma	Neoadjuvant	3	Pneumocystis pneumonia	97.9

CSI, craniospinal irradiation; Gy, gray; V_5 Gy_, volume of organs at risk of receiving more radiation than 5 Gy; M, male; F, female.

**Figure 1:**
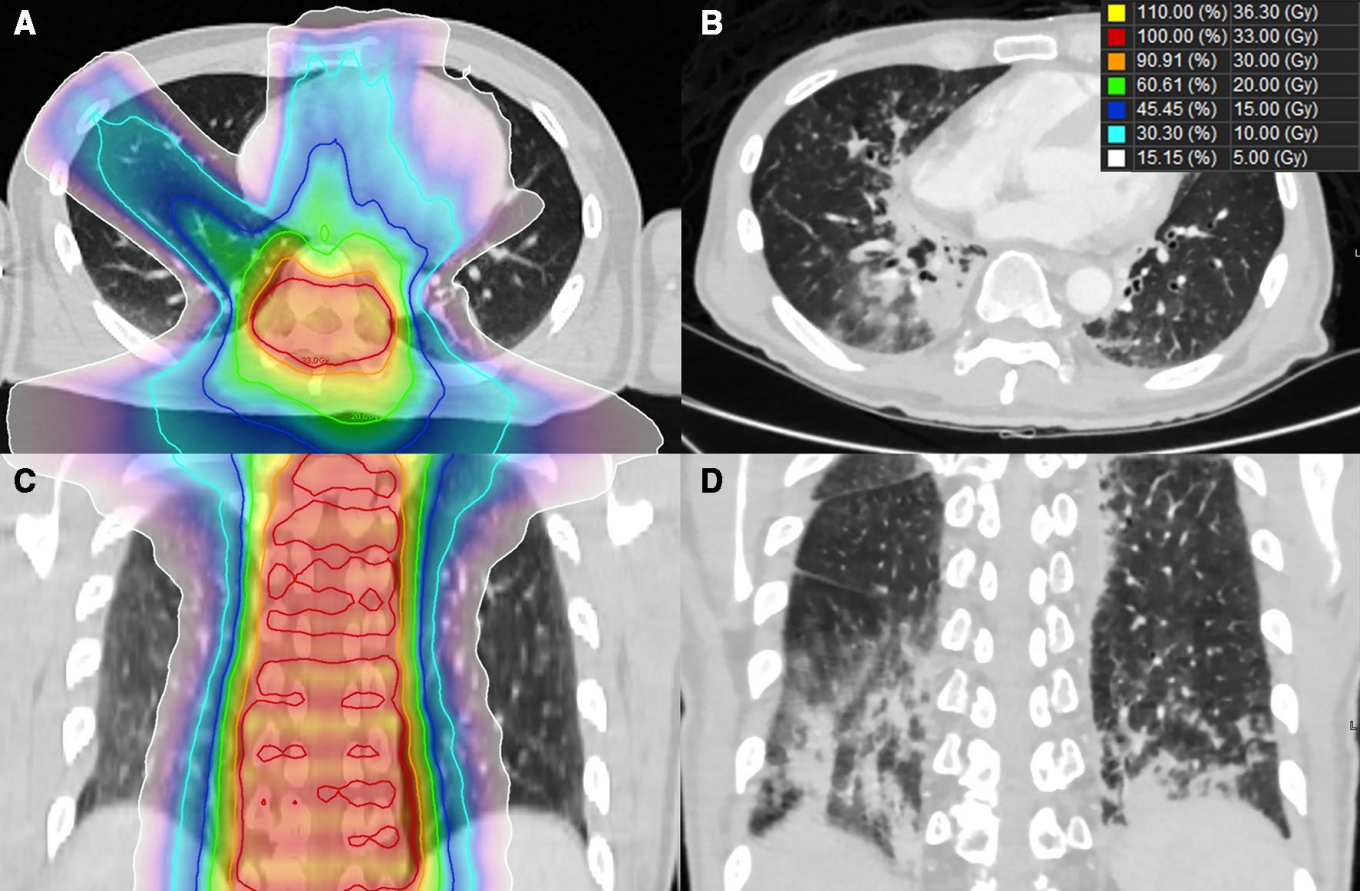
A 52-year-old man in whom grade 2 radiation pneumonitis developed 82 days after initiation of radiotherapy for leptomeningeal carcinomatosis of malignant melanoma. (**A, C**) Dose distribution for craniospinal irradiation and (**B, D**) radiation pneumonitis in computed tomographic image.

The patient diagnosed with grade 2 radiation pneumonitis was a 52-year-old man who underwent HT-CSI for leptomeningeal seeding of malignant melanoma. Radiation pneumonitis occurred 2.7 months after the start of HT-CSI. Bilateral lung V_5 Gy_, V_10 Gy_, V_20 Gy_, and V_30 Gy_ of this patient were 61.5%, 31.4%, 13.0%, 6.0%, and 1.0%, respectively. During and after HT-CSI, he received pembrolizumab. Therefore, it was determined that radiation pneumonitis and pembrolizumab-induced pneumonitis occur together. This patient's dose distribution for HT-CSI and computed tomography (CT) images at the time of diagnosis of radiation pneumonitis are shown in Fig. 1.

Pneumonia occurred median 2.3 months after the start of HT-CSI. In multivariate analysis, intensive systemic treatment (odds ratio (OR) 9.63, 95% confidence interval (CI) 2.07–44.76, *p* = 0.004), immunosuppressive drugs (OR 4.68, 95% CI 1.15–19.0, *p* = 0.031) and bilateral lung V_5 Gy_ ≥ 65% (OR 4.90, 95% CI 1.16–20.77, *p* = 0.031) were identified as independent risk factors for pneumonia (Table 4). Five (5/7, 71.4%) patients diagnosed with pneumocystis pneumonia received intensive systemic treatment, while none of the patients with bacterial pneumonia received intensive systemic treatment. Five (5/7, 71.4%) patients diagnosed with pneumocystis pneumonia and 1 (1/5, 20.0%) with bacterial pneumonia were administered immunosuppressive drugs.

**Table 4:** Prognostic factors for pneumonia in all patients, pediatric patients inclusive.

	Total patients				Pediatric patients			
	Univariate analysis		Multivariate analysis		Univariate analysis		Multivariate analysis	
	OR (95% CI)	*p*-value	OR (95% CI)	*p*-value	OR (95% CI)	*p*-value	OR (95% CI)	*p*-value
Age (Pediatric vs. Adult)	0.64 (0.19–2.17)	0.475			–	–		
Sex (Male vs. Female)	0.35 (0.06–1.93)	0.227			0.64 (0.11–3.65)	0.611		
**Total CSI dose group**								
< 20 Gy vs. 20–36 Gy	1.83 (0.30–11.0)	0.511			1.93 (0.28–13.16)	0.503		
< 20 Gy vs. ≥ 36 Gy	1.29 (0.24–6.90)	0.769			1.50 (0.19–12.15)	0.704		
Dose per fraction for CSI*	1.02 (0.99–1.04)	0.201			1.03 (0.98–1.09)	0.305		
Intensive systemic treatment (No vs. Yes)	5.93 (1.58–22.24)	0.008	9.63 (2.07–44.76)	0.004	4.53 (0.87–23.72)	0.073		
Immunosuppressive drug (No vs. Yes)	3.04 (0.89–10.37)	0.075	4.68 (1.15–19.0)	0.031	4.75 (0.85–26.71)	0.077	13.03 (1.20–141.93)	0.035
Admission during CSI (No vs. Yes)	2.26 (0.58–8.91)	0.242			2.08 (0.36–11.92)	0.409		
Lung V_5 Gy_ (< 65% vs. 65% ≤)	2.67 (0.78–9.08)	0.116	4.90 (1.16–20.77)	0.031	4.00 (0.77–20.73)	0.099	10.17 (1.04–99.79)	0.047
Bone marrow V_5 Gy_ (< 66.9% vs. 66.9% ≤)	1.49 (0.44–5.05)	0.519			3.97 (0.69–22.82)	0.122		

The foreparts of the parentheses were set as the reference groups in the multivariable analysis.

OR, odds ratio; CI, confidence interval; CSI, craniospinal irradiation; Gy, gray; V_5 Gy_, volume of organs at risk of receiving more radiation than 5 Gy.

*Dose per fraction for CSI was treated as a continuous variable.

### Subgroup analysis by age

Mean bilateral lung dose (pediatric vs. adult; median 6.7 Gy vs. 8.1 Gy, *p* = 0.036), bilateral lung V_5 Gy_ (median 48.2% vs. 64.0%, *p* = 0.012), V_10 Gy_ (median 16.3% vs. 24.8%, *p* = 0.032), mean bone marrow dose (median 14.4 Gy vs. 17.7 Gy, *p* = 0.001), bone marrow V_20 Gy_ (median 40.0% vs. 43.0%, *p* = 0.004) and mean heart dose (median 7.4 Gy vs. 8.5 Gy, *p* = 0.001) were significantly higher in adult patients than those in pediatric patients. The beam-on time was significantly longer for adult patients than for pediatric patients (median 518.0 s vs. 616.3 s, *p* = 0.004). A comparison of dosimetric characteristics between pediatric and adult patients is shown in Table 5.

**Table 5:** Comparison of dosimetric characteristics between pediatric and adult patients.

Characteristic	Total (N = 105)		Pediatric (N = 51)		Adult (N = 54)		*p*-value (Pediatric vs. Adult)
	Median	Range	Median	Range	Median	Range	
**Lung**							
Mean dose (Gy)	7.6	3.2–14.7	6.7	3.4–14.7	8.1	3.2–13.3	0.036
V_5 Gy_ (%)	55.2	12.6–100.0	48.2	15.0–100.0	64.0	12.6–99.5	0.012
V_5 Gy_ ≥ 65% (N [%])	39 (37.1)		15 (29.4)		24 (44.4)		
V_10 Gy_ (%)	22.2	0.1–63.3	16.3	0.1–63.3	25.3	0.8–52.4	0.032
V_15 Gy_ (%)	8.2	0.0–33.0	5.7	0.0–33.0	10.6	0.0–28.3	0.099
V_20 Gy_ (%)	3.2	0.0–23.6	1.7	0.0–19.0	4.1	0.0–23.6	0.101
V_30 Gy_ (%)	0.0	0.0–8.5	0.0	0.0–8.5	0.5	0.0–6.8	0.188
**Bone marrow**							
Mean dose (Gy)	16.4	6.8–34.4	14.4	6.8–34.4	17.7	8.8–27.5	0.001
V_5 Gy_ (%)	66.9	33.7–91.3	65.7	46.8–91.3	67.8	33.7–85.6	0.751
V_10 Gy_ (%)	53.5	27.0–79.6	53.1	36.1–79.6	53.5	27.0–68.1	0.902
V_20 Gy_ (%)	42.3	0.0–66.2	40.0	0.0–66.2	43.0	3.5–59.0	0.004
**Heart**							
Mean dose (Gy)	8.0	1.1–22.8	7.4	1.1–22.1	8.5	1.6–22.8	0.001
V_5 Gy_ (%)	93.4	0.0–100.0	87.3	0.0–100.0	95.1	0.0–100.0	0.758
Beam-on time (seconds)	543.4	310.6–964.8	518.0	317.4–964.8	616.3	310.6–924.6	0.004

Gy, gray; V_X Gy_, volume of organs at risk of receiving more radiation than X Gy.

Of the 12 patients diagnosed with pneumonia, 7 (7/51, 13.7%) were pediatric and 5 (5/54, 9.3%) were adult patients. Of the 7 pediatric patients, 4 were diagnosed with pneumocystis pneumonia and 3 were diagnosed with bacterial pneumonia. Of the 5 adult patients, 3 were diagnosed with pneumocystis pneumonia and 2 with bacterial pneumonia. In multivariate analysis for risk factors of pneumonia in pediatric patients, immunosuppressive drugs (OR 13.03, 95% CI 1.20–141.93, *p* = 0.035) and bilateral lung V_5 Gy_ ≥ 65% (OR 10.17, 95% CI 1.04–99.79, *p* = 0.047) were identified as independent risk factors (Table 4). Univariate and multivariate analyses in adult patients showed no independent risk factors associated with pneumonia (see Supplementary Table S2 online).

## Discussion

The results of this study showed that the incidence of grade 2 + pulmonary toxicity was 12.4%, of which 92.3% were in the form of pneumonia. The risk of developing pneumonia increased as immunosuppressive drugs were used, the longer the systemic treatment was received, and the wider the lung was irradiated with low doses.

HT can solve the complex junction problem, which was pointed out as a disadvantage of 3D-CRT and create a homogenous dose distribution by delivering image-modulated beam helically during CSI, which requires treatment of extended volume in the cranio-caudal direction^12^. In addition, HT is more comfortable (both prone or supine) for the patient during treatment and allows for daily verification using megavoltage CT (MVCT) at each fraction^13^.

However, helical delivery of beams can cause the disadvantage of low dose exposure to a wide range of normal tissues. Due to the nature of HT, there have been concerns about lung toxicity caused by low doses, and several related studies have been published. Of these, the largest number of studies were conducted on patients who received RT to the lung lesions directly. In these studies, 27–70% of patients who received RT using HT for lung cancer were diagnosed with grade 2 + pulmonary toxicity, and bilateral lung V_5 Gy_ was found to be a significant predictive factor of pulmonary toxicity, similar to our findings^14–16^. In particular, most of these pulmonary toxicity-related studies set the cut-off value of lung V_5 Gy_ at 65–67.5%, and our findings also showed a significant difference in pulmonary toxicity based on 65% of lung V_5 Gy_. There is also a concern that secondary malignancy may appear more than other RT-induced side effects owing to the widely irradiated low dose. However, there is no study showing clear evidence for secondary malignancy in HT, and although a small number of patients were examined, there were also results that HT did not induce the greater likelihood of secondary malignancy^17,18^.

Another disadvantage of HT is the long beam-on time. According to dosimetric comparative studies between volumetric modulated arc therapy (VMAT) and HT, the beam-on time was approximately two times longer in HT than in VMAT^19^. Especially, HT-CSI has a longer beam-on time because of its wider treatment range than that for other treatment sites; thus, intrafractional movement is likely to occur.

In addition, studies on the feasibility of CSI using VMAT have been recently published^20–22^. As a result of dosimetric comparison, HT was superior to VMAT in planning target volume (PTV) conformity and homogeneity and in organs at risk sparing. Further, lung V_5 Gy_ had a median value of 60–70%, and it was higher than the 55.2% of our study. The main toxicity in CSI using VMAT was hematologic toxicity and showed an incidence rate similar to 3D-CRT. Further studies are needed because studies on CSI using VMAT have relatively fewer patients and a shorter follow-up period than HT-CSI studies.

Some studies analyzed the pulmonary toxicity in patients who received HT-CSI, one of which reported that none of 18 pediatric patients who received HT-CSI were diagnosed with symptomatic acute radiation pneumonitis^23^. Öztunali et al. reported that 2 out of 43 patients aged 7 on average (range 1–56) who received HT-CSI for medulloblastoma were diagnosed with grade 1 pneumonitis and 1 was diagnosed with grade 3 pneumonitis^24^. As these studies showed, all pulmonary toxicity studies in patients who received CSI were mostly conducted on pediatric patients. In both studies, the incidence of radiation pneumonitis was lower than that of the study directly treating lung lesions. The incidence of grade 2 + pulmonary toxicity in our study was 12.4%, but among them, pure radiation pneumonitis comprised one case, with the rest being pneumonia.

One patient with radiation pneumonitis in this study had received pembrolizumab during and after HT-CSI. The relationship between immune checkpoint inhibitor (ICI) and pneumonitis has been reported in several studies, with an incidence rate of 3–5%^25,26^. There is some overlap between the toxicity associated with RT and the toxicity associated with an ICI. One study showed an increase in the incidence of immune-related adverse events of any grade, if the dose is more than 2 Gy per fraction^27^. In the development of pneumonitis, the relationship between low-dose irradiation to the lungs and pembrolizumab is yet to be identified, only relevant case reports exist^28^, and further investigation is needed.

Of the types of pneumonia, cryptogenic organizing pneumonia and pneumocystis pneumonia are particularly associated with RT^29–31^. Cryptogenic organizing pneumonia is a form of idiopathic interstitial pneumonia, and inflammation of the bronchioles (bronchiolitis) and surrounding tissue in the lungs^32^. Cryptogenic organizing pneumonia was known to be caused by interactions between the infection, RT, and immune systems^29^. Among the several factors, RT was thought to play a vital role in the development of cryptogenic organizing pneumonia by causing indirect lung injury by the autoimmune process rather than by direct lung injury^30^. In our study, there were 5 patients with bacterial pneumonia, which is believed to have occurred in the same mechanism as cryptogenic organizing pneumonia. Unlike RT, the relationship between chemotherapy and cryptogenic organizing pneumonia is rarely known, and only case reports have been published^33^. None of the patients diagnosed with bacterial pneumonia in our study received intensive systemic treatment, and 3 (60%) patients did not receive chemotherapy, or received chemotherapy for less than two months; therefore, the connection between bacterial pneumonia and chemotherapy is expected to be low.

Pneumocystis pneumonia is an opportunistic infection caused by a fungus called *Pneumocystis jirovecii*. Its development should be considered in not only immunocompromised patients, but also in patients undergoing intensive chemotherapies and immunotherapies, organ transplantation, or corticosteroid treatment^34–36^. No research has so far shown that RT is an independent risk factor for pneumocystis pneumonia, but studies have shown that there is a risk of opportunistic infection due to lymphopenia, which occurs when RT is combined with chemotherapy or steroid^31^. Lee et al. also reported that prolonged high-dose steroid therapy and concurrent chemoradiotherapy were risk factors for pneumocystis pneumonia development among patients with lung cancer^37^. Of the patients diagnosed with pneumocystis pneumonia in our study, 71.4% received intensive systemic treatment and immunosuppressive drugs, respectively, and all 7 patients diagnosed with pneumocystis pneumonia received either intensive systemic treatment or immunosuppressive drugs.

In both types of pneumonia, RT can be a risk factor, and our study showed a relationship between pneumonia and bilateral lung V_5 Gy_. However, no previous literature has found a direct link between low dose exposure to lung and pneumonia, so further research is needed.

We divided patients into pediatrics and adults, and performed subgroup analysis for each group, with significantly higher dosimetric parameters including bilateral lung V_5 Gy_ in adult patients. This is estimated to be due to a relatively higher prescription dose for CSI in adult patients than in pediatric patients (median 36.0 Gy vs. 23.4 Gy). Subgroup analysis of pediatric patients found that immunosuppressive drugs and bilateral lung V_5 Gy_ were risk factors associated with pneumonia. Immunocompromised children are at risk of pneumonia due to opportunistic pathogens, especially prolonged corticosteroid therapy was found to be a risk factor in pneumocystis pneumonia in pediatric patients^38^. Chemotherapy, which causes bone marrow insufficiency, is also a principal factor in causing pneumonia in pediatric patients^39^. In our study, intensive systemic treatment was not found to be statistically related to pneumonia in pediatric patients, but all pediatric patients diagnosed with pneumonia had received systemic treatment for more than 2 months. Finally, as in the case of overall patients, there was no related literature that revealed the association between pneumonia and bilateral lung V_5 Gy_ in pediatric patients.

One of the limitations of this study was that due to its retrospective nature, there was uncertainty in assessing subjective symptoms that may result in an underestimation of symptomatic pulmonary toxicity. In addition, since all patients who received HT-CSI were analyzed, tumor and treatment characteristics are heterogeneous. Moreover, although the pathophysiology of bacterial pneumonia and pneumocystis pneumonia differ, two types of pneumonia were grouped and analyzed at once. Lastly, since pneumonia is caused by relatively various factors compared to radiation pneumonitis, and the patient to be analyzed also has various treatment and clinical-related factors that can cause pneumonia, there may be confounding factors. Nevertheless, the strength of this study is that among the studies that tried to analyze toxicity in HT-CSI, ours had a large amount of patient data, including 50 or more pediatric and adult patients, respectively. In addition, this is the first study to analyze pulmonary toxicity by subdividing it into radiation pneumonitis and pneumonia, and to suggest the relationship between a low dose irradiated to the lungs and pneumonia. Finally, this study tried to apply the dosimetric factor to the clinical field by analyzing the differences in dosimetric characteristics according to grade 2 + pulmonary toxicity or pediatric/adult.

In conclusion, the findings of this study suggest that HT could be a safe treatment modality with tolerable pulmonary toxicities in terms of CSI. The incidence rate of grade 2 + radiation pneumonitis was 1.0%, and 11.4% of total patients were diagnosed with pneumonia caused by multiple factors. Intensive systemic treatment, immunosuppressive drugs, and bilateral lung V_5 Gy_ ≥ 65% were significantly associated with pneumonia. In pediatric patients, immunosuppressive drugs, and bilateral lung V_5 Gy_ had a greater effect. If any of the above risk factors are present among patients receiving HT-CSI, close follow-up should be considered for proper management of pneumonia. In addition, when planning HT-CSI, it is recommended that the low dose irradiated to the lung be as low as possible, especially V_5 Gy_ should be the most concerned.

## Materials and methods

### Patient selection

HT-CSI (Accuray, Sunnyvale, CA, USA) has been utilized in our institution since 2014. Patients who received HT-CSI between January 2014 and December 2019 were screened (n = 106). Additionally, our institution has also utilized one of the newest generation of tomotherapy delivery systems (Radixact™: Accuray, Sunnyvale, CA, USA) for CSI since November 2018. Patients who received CSI using Radixact™ between November 2018 and December 2019 were also screened (n = 17). Patients who received CSI combined with 3D-CRT (n = 5) or could not complete RT (n = 13) were excluded. Finally, 105 patients were included in our cohort.

This study was approved by the Severance Hospital institutional review board (No. 4–2021-0555). The requirement for informed consent was also waived by the Severance Hospital institutional review board because of the retrospective nature of this study. This study was conducted in accordance with the 1964 Declaration of Helsinki and its later amendments. All methods were carried out in accordance with relevant guidelines and regulations.

### Radiotherapy

All patients underwent simulation CT with 3- or 5-mm slice thickness and intravenous contrast. Thermoplastic masks and Vac-Lok cushion (Blue BAG, Elekta, Stockholm, Sweden) were used for immobilization. Clinical target volume (CTV), PTV, and organs at risk were contoured on simulation CT images using MIM software (MIM Software Inc., Cleveland, OH, USA). CTV for the whole brain and spinal canal plus 3-mm margin were contoured. PTV was generated by expanding the CTV by 3-mm margin at the brain, 5-mm margin at C1-T7 spine level, 7-mm margin at T8-T12 spine level, and 10-mm margin at L1 spine-sacral level. The whole brain, spinal canal, and organs at risk are contoured according to the European Society for Paediatric Oncology (SIOPE) guidelines^40^. TomoTherapy (Accuray, Sunnyvale, CA, USA) or Precision (Accuray, Sunnyvale, CA, USA) software were used for intensity-modulated RT (IMRT) plans. The medical dosimetrists set the following three major parameters: a field width of 2.5–5.0 cm, a pitch of 0.29–0.45, and a modulation factor of 1.4–2.7. PTV coverage was evaluated using the percentage of PTV covered by ≥ 95% of the prescribed dose, with a goal of ≥ 95%. The dose constraints for organs at risk followed the criteria presented in the Quantitative Analysis of Normal Tissue Effects in the Clinic: lung mean dose ≤ 20 Gy, V_20 Gy_ ≤ 30%, and heart mean dose ≤ 26 Gy^41^.

For daily treatment, the patient’s posture was fixed with thermoplastic masks and the Vac-Lok cushion was used when simulation CT was performed^42^. MVCT obtained from the zygomatic arch to the level of the C4 spine was automatically registered in the simulation CT image. After automatic registration, if necessary, the couch was moved to the correct setting in the X, Y, and Z directions at the discretion of the radiation therapist. Post-treatment MVCT ranging from the levels of T12 to L4 was obtained after each fractional treatment was delivered. Because MVCT was acquired at the C spine and L spine levels, no imaging dose was added to the lungs, and only a therapeutic dose could be given.

### Follow-up and assessment of pulmonary toxicity

After RT, patients were followed up by surgeons, oncologists, and radiation oncologists, once monthly then every 3 months for the first 2 years, every 6 months for 1 year, then yearly thereafter. Pulmonary toxicity was diagnosed based on the patient's subjective symptoms and imaging findings. Any grade 2 + pulmonary toxicities (radiation pneumonitis or pneumonia) were evaluated based on the Common Terminology Criteria for Adverse Events version 5.0. Grade 2 pulmonary toxicity was defined by symptomatic aspects, as limiting instrumental activities of daily living, and it required medical intervention.

### Statistical analysis

To analyze factors related to grade 2 + pulmonary toxicities, logistic regression analysis was used. The following variables were used: age (pediatric, adult), sex (male, female), total CSI dose group (< 20 Gy, 20–36 Gy, ≥ 36 Gy), dose per fraction for CSI, intensive systemic treatment, immunosuppressive drugs, admission during CSI, and dosimetric factors (bilateral lung V_5 Gy_, bone marrow V_5 Gy_). Adult and pediatric patients were defined as patients above and below the age of 20 years, respectively. Intensive systemic treatment was defined as systemic treatment administration before, during, and after HT-CSI.

An independent t-test was used to compare the doses irradiated to organs at risks in pediatric and adult patients, and to examine the relationship of dose difference in both groups, and the incidence of pulmonary toxicity.

For multivariate analysis, a backward stepwise selection procedure was adopted. P-values lower than 0.05 were considered statistically significant. Statistical analysis was performed using IBM SPSS, version 25.0 (IBM Corp., Armonk, NY, USA).

## Supplementary Information

Supplementary Information.

## Data Availability

There are no restrictions on the availability of materials or information. The datasets generated during and/or analyzed during the current study are available from the corresponding author on reasonable request.
