# Supplementary material for: Pulmonary toxicity of craniospinal irradiation using helical tomotherapy
Source: Sci Rep. 2022 Feb 25;12:3221. doi: 10.1038/s41598-022-07224-1 (PMC8881492; doi:10.1038/s41598-022-07224-1)
Supplement: Supplementary file 1 — Supplementary Information. [file 41598_2022_7224_MOESM1_ESM.docx]

**Supplementary Table S1.** Dosimetric characteristics of patients according to the presence of grade 2+ pulmonary toxicities

|  | Pulmonary toxicity | | | | |
| --- | --- | --- | --- | --- | --- |
|  | Grade 0 or 1 | | Grade 2+ | | p-value |
|  | Median | Range | Median | Range |  |
| Lung |  |  |  |  |  |
| Mean dose (Gy) | 7.6 | 3.2-14.7 | 7.8 | 4.2-13.3 | 0.607 |
| V_5 Gy_ (%) | 53.9 | 12.6-100.0 | 65.6 | 26.2-99.8 | 0.304 |
| V_5 Gy_≥65% | 23.6 | 0.1-63.3 | 22.2 | 1.9-52.4 | 0.622 |
| V_10 Gy_ (%) | 8.2 | 0.0-33.0 | 8.2 | 0.0-28.1 | 0.840 |
| V_15 Gy_ (%) | 3.3 | 0.0-23.6 | 2.4 | 0.0-16.2 | 0.814 |
| V_20 Gy_ (%) | 0.1 | 0.0-8.5 | 0.0 | 0.0-6.3 | 0.864 |
| V_30 Gy_ (%) |  |  |  |  |  |
| Bone marrow | 16.0 | 6.8-34.4 | 16.0 | 8.4-23.1 | 0.982 |
| Mean dose (Gy) | 66.3 | 46.8-91.3 | 69.4 | 33.7-78.8 | 0.824 |
| V_5 Gy_ (%) | 53.0 | 34.6-79.6 | 55.8 | 27.0-69.3 | 0.921 |
| V_10 Gy_ (%) | 42.1 | 0.0-66.2 | 43.5 | 0.0-58.6 | 0.935 |
| V_20 Gy_ (%) |  |  |  |  |  |
| Heart | 8.0 | 1.6-22.8 | 8.1 | 1.1-22.1 | 0.783 |
| Mean dose (Gy) | 91.5 | 0.0-100.0 | 95.0 | 0.0-100.0 | 0.758 |

***Abbreviations***: Gy, gray; V_X Gy_, volume of organs at risk of receiving more radiation than X Gy

**Supplementary Table S2.** Prognostic factors for pneumonia in adult patients

|  | Univariate analysis | |
| --- | --- | --- |
|  | OR (95% CI) | p-value |
| Sex (Male vs. Female) | 0.28 (0.03-2.71) | 0.274 |
| Total CSI dose group |  |  |
| <20 Gy vs. 20-36 Gy | 0.00 (0.00-0.00) | 0.999 |
| <20 Gy vs. ≥36 Gy | 0.00 (0.00-0.00) | 0.999 |
| Dose per fraction for CSI* | 1.01 (0.99-1.04) | 0.378 |
| Intensive systemic treatment (No vs. Yes) | 0.00 (0.00-0.00) | 0.999 |
| Immunosuppressive drug (No vs. Yes) | 2.82 (0.43-18.57) | 0.280 |
| Admission during CSI (No vs. Yes) | 2.76 (0.29-26.55) | 0.380 |
| Lung V_5 Gy_ (≤65% vs. 65%<) | 2.00 (0.31-13.06) | 0.469 |
| Bone marrow V_5 Gy_ (<66.9% vs. 66.9%≤) | 0.50 (0.08-3.27) | 0.469 |

* Dose per fraction for CSI was treated as a continuous variable

***Abbreviations***: OR, odds ratio; CI, confidence interval; CSI, craniospinal irradiation; Gy, gray; V_X Gy_, volume of organs at risk of receiving more radiation than X Gy

**Supplementary Figure S1.** Dose distribution for craniospinal irradiation by (A) three-dimensional conformal radiotherapy versus (B) helical tomotherapy. The difference between low-dose irradiated volume of both lungs can be compared visually.

**
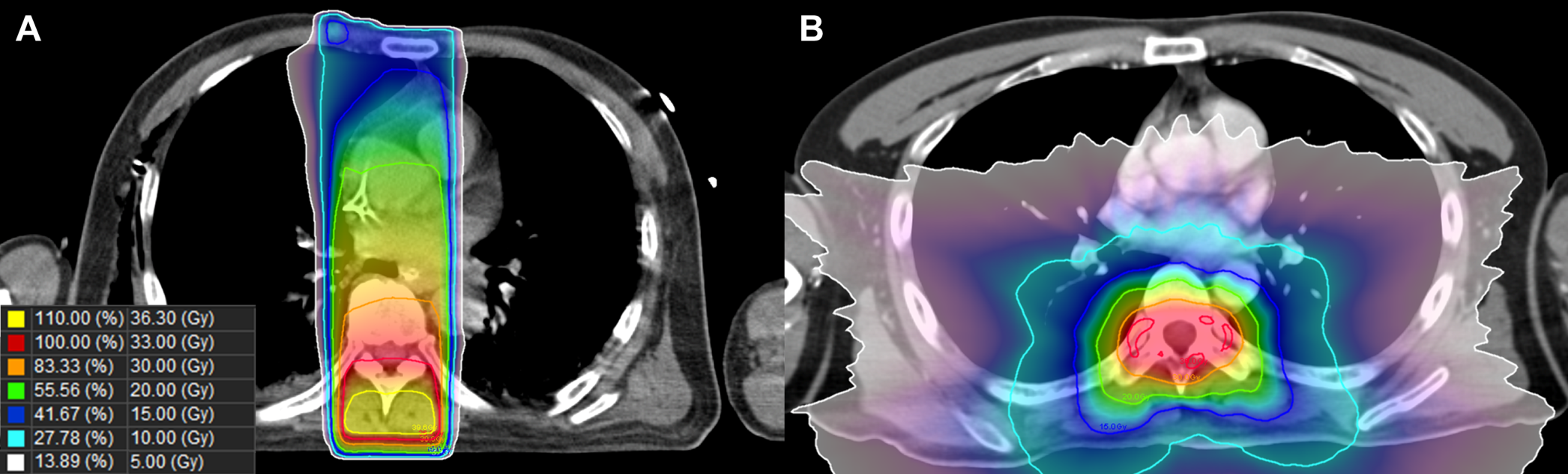
**
